# Lebanon Biogeography Outlined by Tree and Shrub Species Distribution Pattern

**DOI:** 10.1002/ece3.71161

**Published:** 2025-03-19

**Authors:** Jean Stephan, Youmna Hammoud, Melissa Korban, Ignacio Ferro

**Affiliations:** ^1^ L2GE, Department of Life and Earth Sciences, Faculty of Sciences Lebanese University Fanar Lebanon; ^2^ Laboratorio de Ecología Evolutiva y Biogeografía (LEEB)—Instituto de Ecorregiones Andinas (INECOA)—Consejo Nacional de Investigaciones Científicas y Técnicas (CONICET) Universidad Nacional de Jujuy (UNJu) Jujuy Argentina

**Keywords:** biogeography, chorotypes, dissimilarity indices, distributional patterns, ecological niche modeling, elevational gradient, species distribution, transition zones

## Abstract

To delineate the biogeographic structure of Lebanon based on native tree and shrub species distribution using ecological niche modeling and dissimilarity indices. Lebanon, Middle East, and Mediterranean basin (34° N–35° W). We compiled a species list of Lebanon phanerophytes and used ecological niche models (ENM) to obtain surface distributions for each species. Then we defined groups of species with overlapping whole distributional ranges (global chorotypes) and significantly similar distributions within Lebanon (regional chorotype). To evaluate spatially grouped sets of species, we mapped the predicted distribution of species belonging to each chorotype. We measured the biogeographic distinctness amongst them using turnover and nestedness indices. Finally, we identified biogeographic transition zones based on the geographical mixture of chorotypes within Lebanon. We grouped the 60 species into nine global and five regional chorotypes. The chorotypes encompassing more than half of all species were rather nested and occurred on the western side of the country at low to middle elevations of Mount Lebanon (Mediterranean and Euro‐Siberian chorotypes). The most dissimilar chorotypes occurred in the northeastern part of the country on the Anti‐Lebanon mountains (Irano‐Turanian) and the southern lowland (Saharo‐Arabian). We found a hotspot of species richness at intermediate elevation on the northwestern slopes of the Lebanon range and a biogeographic transition zone at high elevation and the eastern slopes of Mount Lebanon, as well as the southern Anti‐Lebanon mountains. Species distribution models allowed us to overcome gaps in occurrence data when studying biogeographical delineation. The first biogeographical structure of Lebanon was generated. The western part of Lebanon belongs to the (East) Mediterranean region, and the northeastern part to the Irano‐Turanian region. The remaining areas are part of biogeographical transitional zones. The few endemic trees are characteristics of these transition zones.

## Graphical abstract

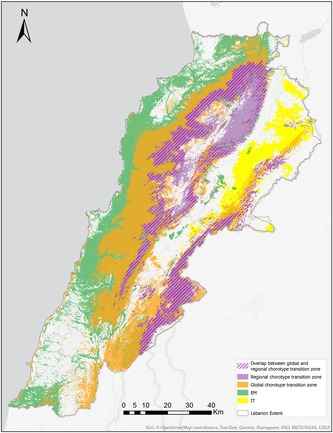

## 1 Introduction

Biogeographers have long divided the earth's surface into relatively homogeneous regions of species composition and described the transition zone as intergradation at the boundaries of adjacent regions. The restriction of plant and animal species to particular areas of the world, namely endemics, allowed biogeographers to divide the planet into different biogeographic regions (Schmid et al. 1987; Moreira‐Muñoz 2007; Holt et al. 2013; Abdelaal et al. 2020). The geographic distribution of a set of species is necessarily nested into its genus, family, and order or class in a hierarchical classification reflecting genealogical relationships. This led to a hierarchical classification of biogeographical regionalization where realms contain regions, which include nested dominions, provinces, and districts. Certainly, Takhtajan (1986) proposed a hierarchical biogeographical system for plants, based on levels of endemism, to distinguish biogeographical units. Alternatively, Kreft and Jetz (2010) defined a framework for delineating biogeographical regions based on areas with similar species composition. Either, based on endemism or similarity, biogeographical areas appear as rather discrete geographical units, and a biogeographic transition zone involves the passage from one to another in the geographical space (Ferro 2024). The European Environmental Agency defines a biogeographical region as an “Area of similar character in terms of the biota (fauna & flora) present in it. Each biogeographic region is based on similarity of composition in terms of the systematics (and hence evolutionary history) of the biota. The extent and boundaries of each region have been determined by changes in climate and the movement of continents, and accompanying changes in the physical and climatic barriers to migration,” sensitize more than three centuries of intense debates on biogeographical regionalization (https://www.eea.europa.eu/help/glossary/eea‐glossary/biogeographical‐region).

**FIGURE 1:**
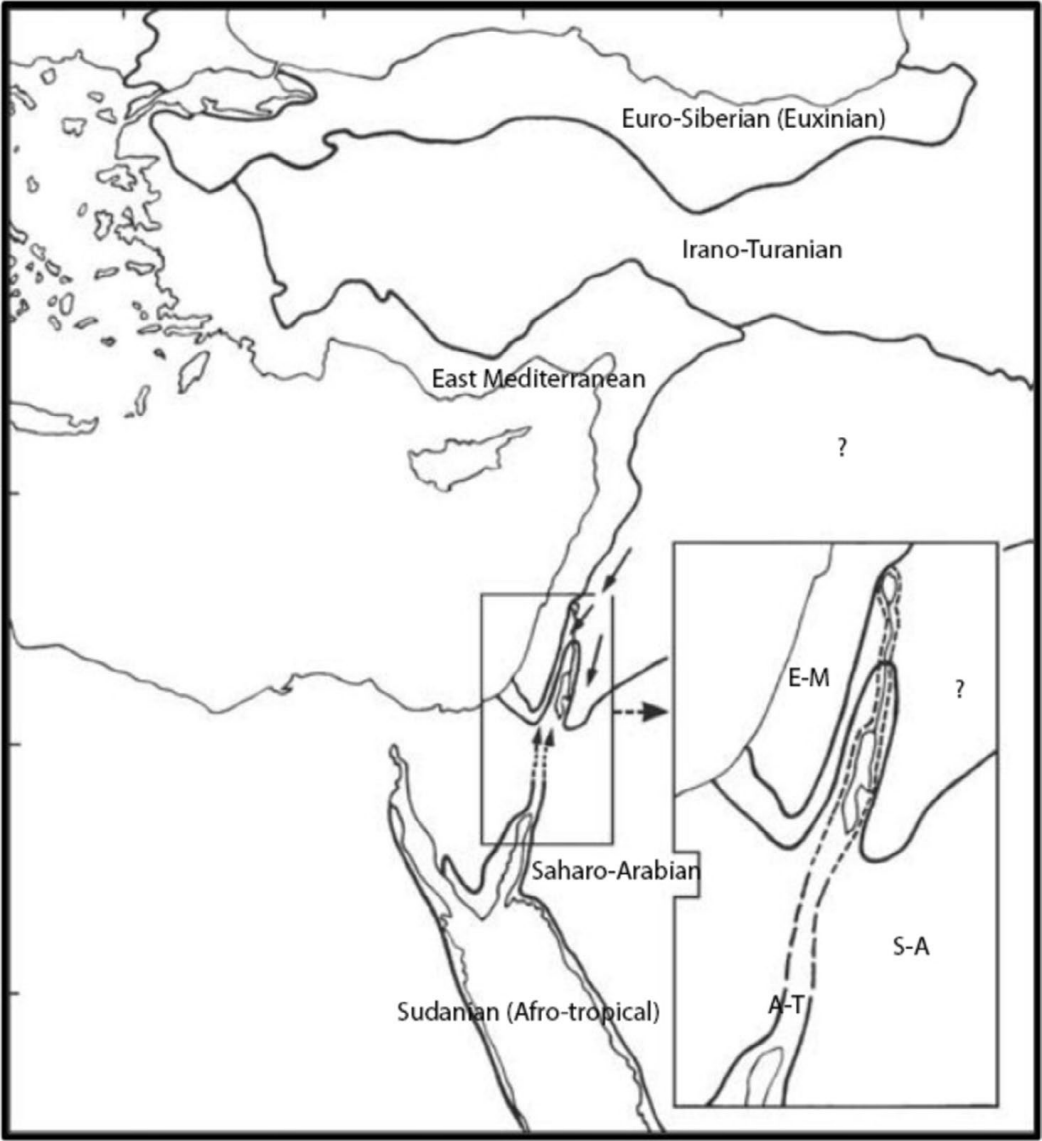
The biogeographical regions in the East Mediterranean Basin (source Blondel et al. 2010).

Morrone (2004) defined biogeographical transition zones as the boundaries between biogeographical regions, representing areas of biotic overlap promoted by historical and ecological changes that allowed the mixture of taxa belonging to different biotic components. Hence, in transition zones, environmental conditions and ecological factors allow both the co‐occurrence of biotic components from different geographical units, but also limit their distribution further one into the other (Ferro and Morrone 2014). Notoriously, biogeographic transition zones frequently coincide with the presence of strong gradients in the physical environment that prevent the dispersal of taxa and maintain taxonomic differentiation between adjacent regions. The restriction for such biotic assemblages may be a strong environmental gradient or ‘ribbons’ of unsuitable habitats (Glor and Warren 2011). For example, sharp environmental gradients may occur in those transition zones associated with mountain ranges, such as the Alborz Mountains separating the Euro‐Siberian (Hyrcanian) from the Irano‐Turanian region (Djamali et al. 2008). Furthermore, paths of unsuitable habitats may have an underlying environmental gradient but are not necessarily sharp. A good example may be the Sinai Peninsula or the Syrian Desert separating the Saharo‐Arabian from the Mediterranean or Irano‐Turanian regions. Yet, some delineations are less evident, such as the transition between the Mediterranean and Euro‐Siberian regions in the Iberian and Italian peninsulas (Blasi et al. 1999; Blondel et al. 2010). Besides, biogeographical units may also be differentiated according to climatic, geographic, physiologic, or other approaches. For delineating biomes, researchers relied on climatic parameters, topography‐soil parameters, and life forms, which influence the distribution of living beings. On a map, these biogeographical units are frequently enclosed by lines symbolizing the boundaries between adjacent areas (Blasi et al. 1999; Simpson et al. 2009; Blondel et al. 2010; Kreft and Jetz 2010; Djamali et al. 2012). However, rather than straight lines, biotas intergrade into one another as zones with a certain degree of geographic extension when closely observed (Divíšek et al. 2016; Ferro and Morrone 2014). In this regard, Mouterde (1966) could not define a sharp delineation between the Mediterranean area and the Irano‐Turanian in the Levantine countries. To avoid the misleading attribution of “Continental Mediterranean” he suggested a “Syrian climate” with specific vegetation and endemic species for large areas stretching from the Golan Heights and Jabal el Druze in Syria towards the Beqaa Valley in Lebanon, and the northwestern mountains of Syria (Idlib and Aleppo) to the border with Turkey and upper Mesopotamia. Zohary (1973) and later Blondel et al. (2010) noted the importance of this part of the World as a crosswalk between three continents (Asia, Europe, and Africa) with various floristic groups and biogeographical areas (Mediterranean, Euro‐Siberian, Irano‐Turanian, Saharo‐Arabian, and Sudanian), but failed to define an accurate delineation of each (Figure 1). Despite the intertwined floristic groups and biogeographical areas, mainly between the rift valley to the north of the Dead Sea up to Lebanon, some zones remain unattributed to any floristic group (question mark in Figure 1). Yet, a biogeographic map defining the structure of countries like Lebanon has been lacking; the great topographic and environmental heterogeneity in a small expanse, with a remarkable elevation range (0–3000 m asl) and precipitation gradient, with a semi‐arid region in the northeast, means that several details of the biological composition across space may be overlooked in the framework of a continual analysis.

Given that a biogeographical transition zone occurs as contact between two or more regions, it is important to deconstruct the flora or fauna and determine their biogeographical appurtenance to a given region. Delineation of transition zones can be achieved by plotting distribution maps of species occurrences so that sets of species with repetitive distribution can be identified as a pattern and then depict their mixture on geographical grounds. The concept of chorotype is a common approach to denote the biogeographical composition of a species assemblage (Passalacqua 2015; Fattorini 2016). Species with similar geographical ranges can be grouped into chorotypes, and recurrent species co‐occurrences represent their affinity in terms of geographical distribution. The chorotype has been interpreted as assemblages of species with certain ecological requirements that shape their distribution. However, different concepts can be included under the term chorotype. For instance, Fattorini (2015) distinguished between regional and global chorotypes depending on the geographical framework used to analyze species with overlapping ranges. Regional chorotypes involve groups of species with similar distribution at a local scale within a given study area previously defined. In contrast, global chorotypes indicate a group of species with similar overall distribution ranges. Depending on the selected study area, a given species might be classified into different regional chorotypes in the former concept. The latter implicates the whole distribution range and does not vary with the study area under consideration.

Another approach to visualizing patterns of similarities and differentiation in the biotic composition is to use similarity indices, which calculate taxonomic distinctiveness that can then be displayed on maps (Rodrigez and Arita 2004; Rivas‐Martínez et al. 2011; Dapporto et al. 2015). Yet, patterns in taxonomic (dis)similarity may arise from two sources of variation in species composition: the real turnover and the nestedness components, which may lead to divergent results due to differences in species richness among the studied regions (Baselga 2010, 2012; Kreft and Jetz 2010; Mouillot et al. 2013). Species turnover in its narrow sense implicates the replacement of one species by another when moving across regions or localities, whereas the broad sense turnover implicates nestedness, losses, or gains; thus, gradients of species richness, which are subsets of the species assemblages but without a real replacement (Ulrich and Gotelli 2007; Baselga 2010).

The eastern Mediterranean region might exhibit both phenomena. The rugged topography of Taurus, Anti‐Taurus, and Amanus, in southern Turkey and the Lebanon mountain ranges is considered a refuge for the Tertiary floras (Médail and Diadema 2009). Thus, relict species distributions from the glaciation periods thrive in some mountain enclaves with cold and mesic conditions (Awad et al. 2014; Walas et al. 2019; Douaihy et al. 2020; López‐Tirado et al. 2020; Stephan et al. 2020). On the contrary, the sharp decrease and change in biodiversity pattern of richness when moving from the coastal area towards the Syrian Desert might result from changes in bioclimatic zones (Blondel et al. 2010; Bou Dagher‐Kharrat et al. 2018). Despite the importance of the region as a biodiversity hotspot coupled with historical anthropogenic influence, the delineation of the biogeographical divisions and transition zones remains vague in the Levant countries.

In this work, we investigate the biogeography of Lebanon based on the distribution of tree and shrub species. We aim to propose a biogeographical framework of Lebanese territory using predicted distribution maps, chorotypes, and turnover indices to depict biotic assemblages. We seek to delineate the country's biogeographic structure and provide a visual illustration of those transitional areas, as tools for the management and conservation of biological resources.

## 2 Methods

### 2.1 Study Area

**FIGURE 2:**
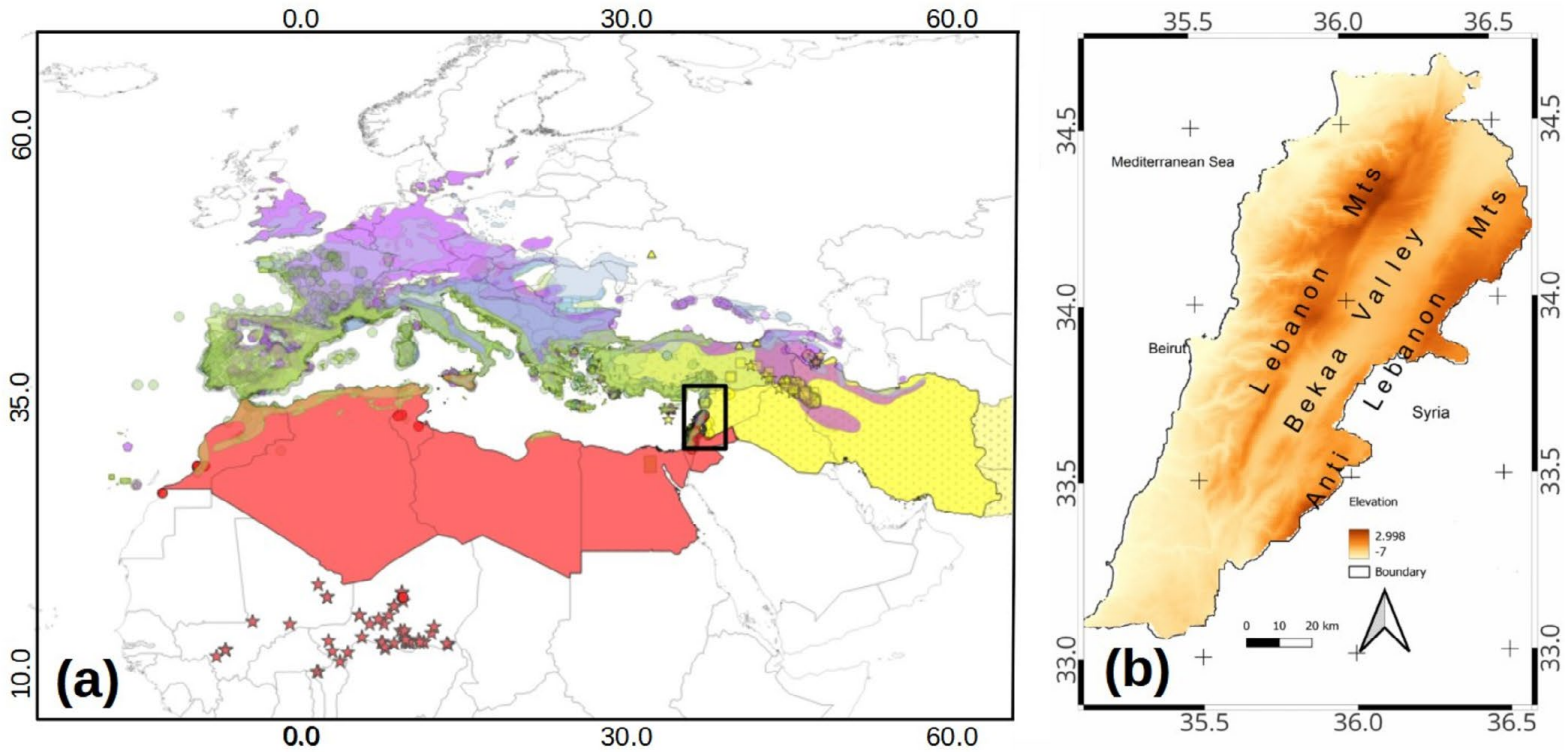
Study area. (a) A simplified scheme of the whole distribution range for some plants in Lebanon (rectangle) according to their expanses into the biogeographical regions of the Mediterranean as defined by Blondel et al. (2010). Green for Mediterranean and East Mediterranean, yellow for Irano‐Turanian, purple for Euro‐Siberian, and red for Saharo‐Arabian species. The list of species per global chorotype is detailed in Table 1. Shapefiles were downloaded from Euforgene (https://www.euforgen.org/) and IUCN (https://www.iucnredlist.org/) in May 2022. (b) Lebanon's extant and geographical features.

The study area corresponds to Lebanon, located in the eastern Mediterranean Sea, with 220 km of shore and about 50 km inland (Figure 2a,b). This 10.452 km^2^ territory has a diverse physical geography. After a narrow coastal strip, the Lebanon mountain range rises parallel to the coast, reaching the highest summits (3000 m above sea level) north. Most of the eastern slopes drain to the Litani and Orontes Rivers in the central Beqaa Valley, framed to the west by the Anti‐Lebanon mountain range with the highest summit above 2800 m, which forms the natural border of the country with Syria (Figure 2b).

Although the climate is typically Mediterranean, the mountain ranges have a remarkable influence on the local climate. The average annual temperature is roughly 20°C on the coast. However, temperature drops with elevation, coupled with a substantial increase in precipitation in the form of snow at higher altitudes, reaching up to 1400 mm on the western slopes of Mount Lebanon. The eastern slopes and the Beqaa Valley are drier due to the rain shadow, with precipitation varying between 200 and 800 mm and higher daily and seasonal temperature differences. These variations result in a high species diversity and natural ecosystems, making Lebanon a biodiversity hotspot (Bou Dagher Kharrat et al. 2018).

According to Blondel et al. (2010), there are species with biogeographic appurtenance to five regions in the study area (Figure 1). The Mediterranean (M) and within it the East Mediterranean (EM), the Irano‐Turanian (IT), The Euro‐Siberian (ES) and The Saharo‐Arabian with intrusions of the Afro‐Sudanian (SA).

The Mediterranean is characterized by a dry summer and a mild, wet winter. Species common to the Mediterranean include *Pinus pinea, Laurus nobilis*, and *Ceratonia siliqua* . The East Mediterranean is distinguished by a longer dry period and the presence of several species considered vicariate to those of the Western Mediterranean, and more acclimated to the climatic and soil conditions, such as *Quercus coccifera subsp. calliprinos, Quercus ithaburensis, Cedrus libani*, and *Abies cilicica*. The relic species of the Euro‐Siberian include species that require cool and humid conditions, such as *Ostrya carpinifolia, Sorbus torminalis*, and *Rosa canina* . The Irano‐Turanian is represented by xeric species that are also adapted to cold winters, such as *Prunus argenta* (*Amygdalus orientalis*), *Prunus microcarpa*, or *Prunus korshinskyi*. The Saharo‐Arabian is characterized by a hot and dry climate with species such as *Ziziphus spina‐christi, Searsia tripartita*, and *Pistacia atlantica* .

### 2.2 Data Set

We included presence point records of tree and shrub species collected in the field all over Lebanon and complemented them with literature records (Abi Saleh et al. 1996; Stephan et al. 2016, 2019, 2020; Hammoud and Stephan 2022; Stephan and Korban 2025). The selected taxa included solely native phanerophytes (60 trees and shrubs out of 120 approximately) having substantial presence point data, allowing accurate and valid predicted areas of distribution through ecological niche modeling. Species with minimal presence points or those difficult to identify or known only from planted specimens and exclusive to riparian zones were discarded to avoid biased results in the analysis (Chao et al. 2004).

### 2.3 Analyses

#### 2.3.1 Ecological Niche Modeling

Our approach relies on distribution maps of 60 tree and shrub species, generated through ecological niche modeling (ENM). The presence points of each taxon were input for modeling distribution with a list of predictors that constitute the environmental variables that contributed to each of the studied taxa (see the list of variables, and their sources in the table in Table A1). We adjusted MaxEnt settings based on the number of presence points and their grouping, environmental range, and distribution extent in Lebanon (Hammoud and Stephan 2022; Merow et al. 2013; Phillips and Dudík 2008). We examined the area under the curve (AUC) to validate the models for the different taxa, and only scores with AUC values above 0.85 were retained (Araújo et al. 2014).

#### 2.3.2 Definition of Chorotypes

In our methodology, we followed the recognition of chorotypes as described by Fattorini (Fattorini 2015). Therefore, we differentiate the similarity between species distribution within Lebanon's extent (the regional chorotypes) from those that cover the whole geographic distribution range (the global chorotypes).

To define global chorotypes, we used literature‐based revision and reconstructed the whole species distributional range using an inductive and recursive process (Di Biase et al. 2021) in which species distributions are mapped, their contours are compared, and species with similar ranges are classified into the same groups. The groups were then named based on their appurtenance to biogeographical regions (Davis 1965–1985; Zohary 1973; Tohmé and Tohmé 2007; Stephan et al. 2020). Global chorotypes were named by their appurtenance to one region, two regions, or three regions. As a result, taxa were integrated into nine global chorotypes.

To detect regional chorotypes, we used the predicted distribution for every species within Lebanon to build a presence‐absence matrix on a square grid of 6 × 6 km. Then we used Baroni‐Urbani & Buser's (1976) index to search for significant similar distribution among species, as described by Olivero et al. (2011) for the detection of chorotypes as fuzzy sets (implemented in ‘RMACOQUI’ package for R). This method, based on a quantitative classification of distributional areas, generated a dendrogram with the agglomerative unweighted pair‐group method using arithmetic averages (UPGMA). Then, the dendrogram is examined to identify branches that exhibit significant positive within‐branch shared distributions and significantly disjunct from adjoining branches. An index of internal homogeneity and distinctness (IH, ranging from −1 to +1) is derived by considering pairwise comparisons of the proportion of significant similarity and significant dissimilarity distributions in every branch. A given branch of the dendrogram is considered a chorotype if IH = 1, or positive and higher than subsequent nested clusters, and statistically significant. Significance is evaluated by comparing the frequency of significant similarities within a tested cluster and the most similar branch of the dendrogram using a G‐test of independence. Finally, a series of fuzzy logic parameters is computed based on the average of similarities between each species distribution and all the distributions in a chorotype. The computation of fuzzy logic parameters allows us to evaluate the degree of membership of any given distribution to every chorotype, the overlap or similarity between chorotypes, and the degree to which a chorotype is included in another one (see Olivero et al. 2011).

#### 2.3.3 Turnover and Nestedness Amongst Chorotypes

To measure the differentiation amongst the chorotypes present in Lebanon, we used a beta‐diversity index. Beta‐diversity or turnover may arise from two sources of variation: replacement (true turnover) or nestedness (richness difference). We calculated the pairwise turnover index using Sorenson and Simpson dissimilarity indices to separate these two components. The first is sensitive to richness differences, while the latter is a strict turnover index independent of richness gradients. This lets us overcome the possible uneven richness amongst chorotypes (Mouillot et al. 2013). Given that in the absence of richness differences, both indices are equal, the nestedness component can be identified as the difference between the Sorenson dissimilarity index and the turnover rate given by the Simpson index (Baselga 2010, 2012).

The three equations of Sorenson dissimilarity (*β*_sor), turnover (*β*_sim) and nestedness (*β*_nest) are displayed in Equations ((1), (2), (3)) along with their respective versions for surface comparison:

(1) $$\beta_{\mathrm{sor}}=\frac{b+c}{2a+b+c}=\frac{\sum_{c}^{b}S_{\text{group}}}{2\overset{c}{\underset{b}{⋂}}S_{\text{group}}+\sum_{c}^{b}S_{\text{group}}}$$

(2) $$\beta_{\mathrm{sim}}=\frac{\min\left(b,c\right)}{a+\min\left(b,c\right)}=\frac{\min S_{\text{group}\;\left(b,c\right)}\;}{\overset{c}{\underset{b}{⋂}}S_{\text{group}}+\min S_{\text{group}\;\left(b,c\right)}}$$

(3) $$\beta_{\text{nest}}=\beta_{\mathrm{sor}}-\beta_{\mathrm{sim}}$$

where *a* is the common (intersection) area between two groups, *b* and c are the total (union) areas for the intersection and one of the two groups of species compared, while max (*b,c*) and min (*b,c*) refer to the group of species showing the maximal and the minimal predicted areas of distribution amongst the two groups. We can then estimate the degree of dissimilarity, turnover, and nestedness amongst pairs of chorotypes and evaluate distinctiveness, non‐overlapping, and the extent of partial overlapping between the chorotypes, amongst the biogeographic units they occupy in Lebanon.

#### 2.3.4 Mapping Species Richness and Biogeographical Transition Zone

To depict species richness gradients of tree and shrub species in Lebanon, we added all species' predicted distribution areas in the country. To distinguish gradients of biogeographical affinities across the studied area, we displayed the variation in local and global chorotypes on the map. Because a biogeographical transition zone is defined as a mixture of species sets with different distribution patterns, we used the number of species belonging to a given chorotype relative to the total number of species in each pixel to depict the combination of species with different biogeographical affinities. To find the places where the maximum mixture of species belonging to different chorotypes occurs, we used their relative frequency to calculate the Simpson index of diversity (Hsim). This index measures the degree of concentration; thus, higher values of Hsim indicate more concentration (homogeneity) and lower values indicate more diversity (heterogeneity). We then used a color scale of similar values to create a map of the heterogeneity of regional and global chorotypes highlighting gradual changes in dominance and hotspots of heterogeneity (transition zone).

## 3 Results

### 3.1 Global Chorotypes

**FIGURE 3:**
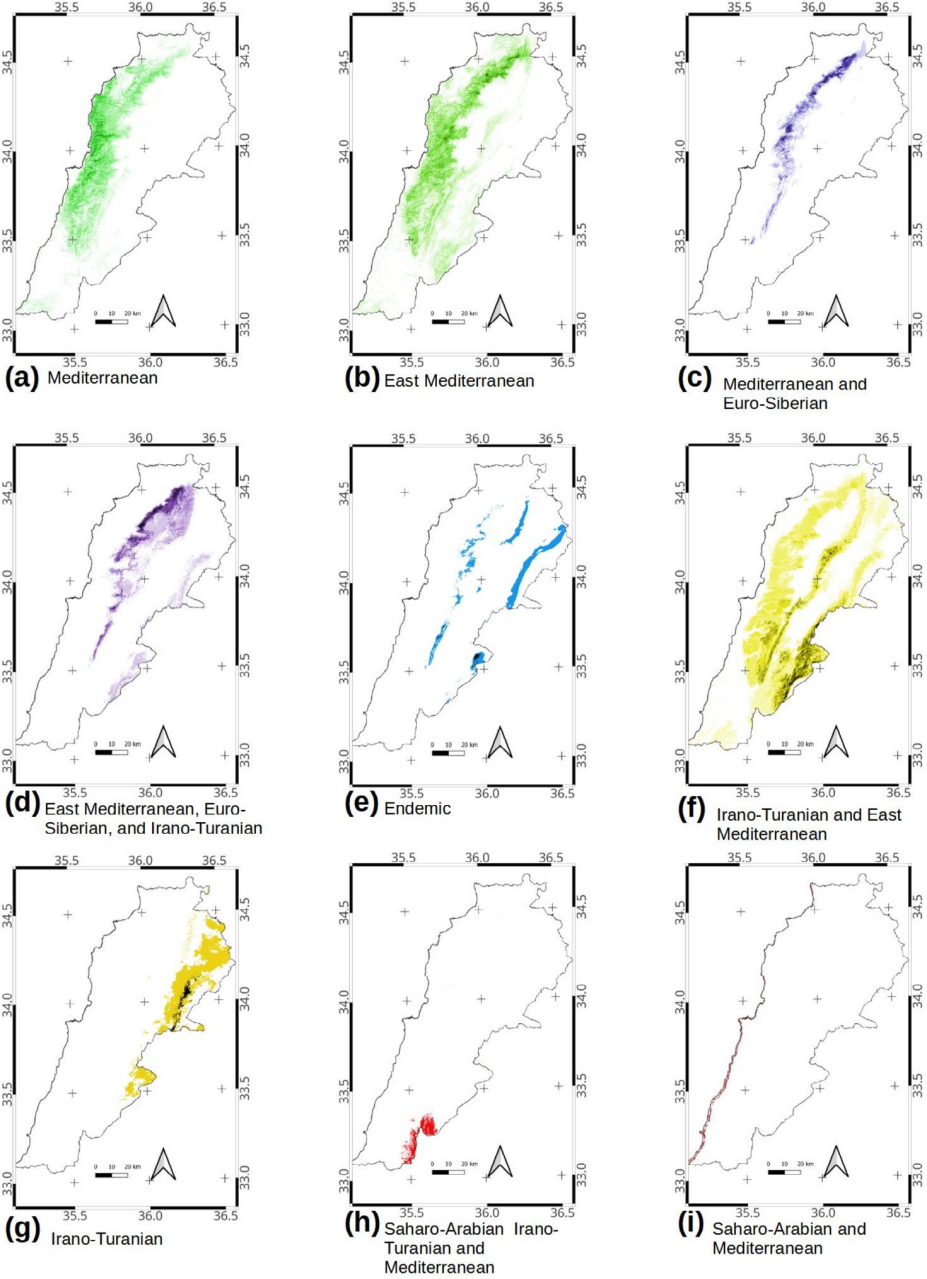
Local distribution of global chorotypes for each group of species within the study area. (a) Mediterranean, (b) East Mediterranean, (c) Mediterranean and Euro‐Siberian, (d) East Mediterranean, Euro‐Siberian, and Irano‐Turanian, (e) Endemic, (f) Irano‐Turanian and East Mediterranean, (g) Irano‐Turanian, (h) Saharo‐Arabian, Irano‐Turanian, and Mediterranean, (i) Saharo‐Arabian and Mediterranean (narrow strip on the coast).

Of the 60 species used for the ecological niche model analysis, half of them (31 species) matched either a Mediterranean (14 species) or Eastern Mediterranean (17 species) global chorotype (Table 1). The predicted maps resulting from the ecological niche modeling of these species revealed that their distribution is mainly restricted to the western (central and northwestern) parts of the country (Figure 3a,b). Despite their similar overall distribution in Lebanon, there were certain differences in the local variation of these two global chorotypes. The species in the Mediterranean chorotype only occur on the western slopes of the Lebanon mountain range and tend to be more frequent at lower elevations (Figure 3a), while the Eastern Mediterranean global chorotype extends to the Anti‐Lebanon mountains and shows high species richness in the middle elevation on the western slopes of the Lebanon mountain range (Figure 3b). The nine species attributed to the Mediterranean and Euro‐Siberian chorotype occur as a narrow strip above 1400 m on the western slopes of Mount Lebanon (Figure 3c). The six species attributed to the East Mediterranean, Euro‐Siberian, and Irano‐Turanian global chorotype, whose global distributions also extend eastwards, occur at even higher altitudes (above 2000 m) of the Lebanon mountain range, on both slopes, and the Anti‐Lebanon mountains (Figure 3d). There are three range‐restricted species representing Lebanon endemics (*Pyrus bovei, Quercus kotschyana*, and *Quercus look*), distributed in Lebanon and Anti‐Lebanon mountains (Figure 3e). The seven species with a distribution range grouped into the Irano‐Turanian and East Mediterranean global chorotype occur at middle to high altitudes of Mount Lebanon and the western slopes of Anti‐Lebanon and Hermon (Figure 3f). The two species that match an Irano‐Turanian distribution (*Amygdalus orientalis* and *Rosa foetida* ) only occur in the eastern part of the country associated with the Anti‐Lebanon range (Figure 3g). Finally, the two species that extend southward to the Saharo‐Arabian region were disjointed within Lebanon. *Pistacia atlantica* was strictly found in the southeastern part of Lebanon down to the Rift Valley, below 600 m (Figure 3h) and *Searsia tripartita* is only found in a very narrow strip on the coastline (Figure 3i).

**TABLE 1:** The studied taxa and their global and regional chorotype appurtenance.

Species	Global chorotype	Regional chorotype
Abies_cilicica	East Mediterranean	C3
Acer_monspessulanum	East Mediterranean, Euro‐Siberian, and Irano‐Turanian	C3
Acer_obtusifolium	East Mediterranean	C4
Acer_tauricolum	East Mediterranean	C3
Amygdalus_orientalis	Irano‐Turanian	C5
Arbutus_andrachne	East Mediterranean	C4
Atadinus_libanoticus	East Mediterranean, Euro‐Siberian, and Irano‐Turanian	C3
Buplerum_fruticosum	Mediterranean	
Calicotome_villosa	Mediterranean	C4
Cedrus_libani	East Mediterranean	C3
Ceratonia_siliqua	Mediterranean	C4
Cercis_siliquastrum	Mediterranean	C4
Colutea_cilicica	Mediterranean and Euro‐Siberian	C3
Cornus_sanguinea	East Mediterranean	C3
Cotoneaster_nummularia	East Mediterranean, Euro‐Siberian, and Irano‐Turanian	C3
Crataegus_azarolus	Irano‐Turanian and East Mediterranean	C4
Crataegus_monogyna	Mediterranean	C3
Cupressus_sempervirens	Mediterranean	C4
Fraxinus_ornus	Mediterranean and Euro‐Siberian	C3
Juglans_regia	Mediterranean and Euro‐Siberian	C3
Juniperus_drupacea	East Mediterranean	C3
Juniperus_excelsa	East Mediterranean, Euro‐Siberian, and Irano‐Turanian	C3
Juniperus_foetidissima	Mediterranean and Euro‐Siberian	C3
Juniperus_oxycedrus	Mediterranean and Euro‐Siberian	C3
Laurus_nobilis	Mediterranean	C4
Lonicera_nummularifolia	East Mediterranean, Euro‐Siberian, and Irano‐Turanian	C3
Malus_trilobata	East Mediterranean	C3
Myrtus_communis	Mediterranean	C4
Ostrya_carpinifolia	Mediterranean and Euro‐Siberian	C3
Phillyrea_media	Mediterranean	C4
Pinus_brutia	East Mediterranean	C4
Pinus_halepensis	Mediterranean	C4
Pinus_pinea	Mediterranean	C4
Pistacia_atlantica	Saharo‐Arabian, Irano‐Turanian and Mediterranean	
Pistacia_eurycarpa	Irano‐Turanian and East Mediterranean	C5
Pistacia_lentiscus	Mediterranean	C4
Pistacia_palaestina	East Mediterranean	C4
Prunus_dulcis	Irano‐Turanian and East Mediterranean	C2
Prunus_korshinskyi	Irano‐Turanian and East Mediterranean	C5
Prunus_microcarpa	Irano‐Turanian and East Mediterranean	C2
Prunus_ursina	East Mediterranean	C3
Pyrus_bovei	Endemic	C5
Pyrus_syriaca	Irano‐Turanian and East Mediterranean	C4
Quercus_calliprinos	East Mediterranean	C4
Quercus_cerris	Mediterranean and Euro‐Siberian	C3
Quercus_infectoria	Irano‐Turanian and East Mediterranean	C4
Quercus_ithaburensis	East Mediterranean	
Quercus_kotschyana	Endemic	C3
Quercus_look	Endemic	C1
Rhamnus_alaternus	Mediterranean	C4
Rhamnus_punctata	East Mediterranean	C4
Rosa_canina	Mediterranean and Euro‐Siberian	C3
Rosa_foetida	Irano‐Turanian	C5
Rosa_phoenicia	East Mediterranean	C4
Searsia_tripartita	Saharo‐Arabian and Mediterranean	
Sorbus_flabellifolia	East Mediterranean, Euro‐Siberian, and Irano‐Turanian	C3
Sorbus_torminalis	Mediterranean and Euro‐Siberian	C3
Spartium_junceum	Mediterranean	C4
Styrax_officinalis	East Mediterranean	C4
Viburnum_tinus	Mediterranean	C4

*Note:* The chorotypes classification is based on the appurtenance of a biogeographic region with an additional group for endemics to Lebanon. The regional chorotypes based on significant distribution range congruence within Lebanon are named according to their geographical location in the country. C1: Southern Mount Lebanon range, C2: Southeastern Lebanon and Anti‐Lebanon range, C3: Western slopes high‐elevation of Mount Lebanon range, C4: Western slopes middle and low‐elevation of Mount Lebanon range, C5: North‐eastern Lebanon and Anti‐Lebanon range.

### 3.2 Regional Chorotypes

**FIGURE 4:**
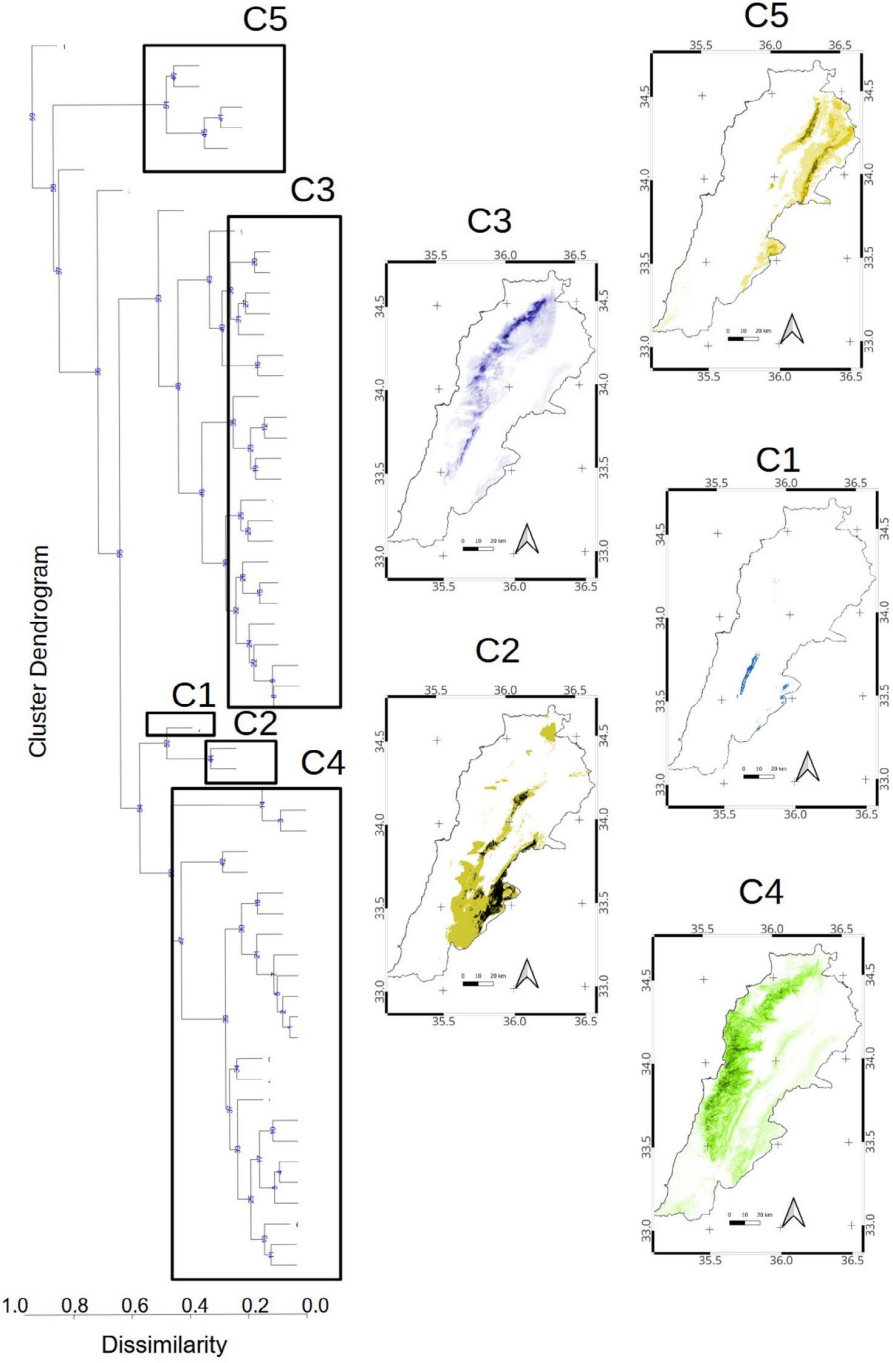
Geographical distribution of regional chorotypes based on significant coincident distributional ranges obtained for the 0.6 × 0.6 km grid analysis within Lebanon. (a) Clusters of significant coincident distributional ranges are indicated by rectangles coded as C1, C2, C3, C4, C5 (b) Species´ geographical distributions belonging to each chorotypical cluster.

We found five regional chorotypes, a set of species with a significant congruent distributional range within Lebanon (Figure 4), which included 56 of the 60 species analyzed in our matrix (Table 1). Chorotype C1, in southern Mount Lebanon, included only *Quercus look*, which is endemic to Lebanon; while C2, which occurs in southeastern Lebanon and the Anti‐Lebanon mountains, grouped two species: *Prunus dulcis* and *Prunus microcarpa* (Figure 4b). The two richest chorotypes, C3 and C4, grouped 24 species into each one. Both were mainly distributed in the western part of the country, but C3 grouped those species occurring at higher altitudes in a narrow strip on the western slopes of Mount Lebanon, while C4 included lowland and middle‐elevation species on the western slopes of the Mount Lebanon range (Figure 4, Table 1). The regional chorotype C5 was the most dissimilar and grouped five species with significantly similar distributions (*Pyrus bovei, Prunus argenta, Prunus korshinskyi, Pistacia eurycarpa*, and *Rosa foetida* ), which all occur in the eastern half of the country associated with the northern eastern slopes of the Lebanon range and the Anti‐Lebanon Mountains (Figure 4).

### 3.3 Chorotype Expanse, Dissimilarity, and Nestedness

The chorotype belonging to the Eastern Mediterranean has a wider range than the circum‐Mediterranean chorotype in Lebanon. Both chorotypes are the richest in species and together group half the species in this analysis. The Eastern Mediterranean‐Irano‐Turanian chorotype also occupies a wide area of the country, but it is just grouped into seven species (see Table 2). On the other hand, the smallest surface is occupied by the Saharo‐Arabian chorotype with a few species reaching the south‐eastern and south‐west lowlands of the country. While the Irano‐Turanian chorotype has a modest distribution surface (87,328 ha) and is represented by two species, it is markedly dissimilar to other chorotypes (Figure 3g, Table 3). Considering the results of turnover and nestedness values amongst chorotypes, it is evident that the maximal dissimilarity and turnover rates occur between the Irano‐Turanian chorotype as well as the Saharo‐Arabian compared with the rest of the chorotypes present in the country (Table 3).

**TABLE 2:** Global and regional chorotypes species richness and potential area of distribution (ha).

Global chorotype	Species richness	Area (ha)	Regional chorotype	Species richness	Area (ha)
M	15	310.32	C1	2	192.53
EM	16	369.17	C2	1	8.07
M‐ES	9	122.75	C3	24	240.69
EM‐ES‐IT	6	132.01	C4	24	474.09
END	3	52.61	C5	5	166.2
IT‐EM	7	416.78	—	—	—
IT	2	87.33	—	—	—
SA‐IT‐M	1	7.77	—	—	—
SA‐M	1	4.28	—	—	—

*Note:* Global Chorotype EM, East Mediterranean; EM‐ES‐IT, East Mediterranean, Euro‐Siberian, and Irano‐Turanian; END, Endemic to Lebanon; IT, Irano‐Turanian; IT‐EM, Irano‐Turanian and East Mediterranean; M, Mediterranean; M‐ES, Mediterranean and Euro‐Siberian; SA‐IT‐M, Saharo‐Arabian, Irano‐Turanian and Mediterranean; SA‐M, Saharo‐Arabian and Mediterranean. Regional Chorotypes C1, Southern Mount Lebanon range C2, Southeastern Lebanon and Anti‐Lebanon mountains, C3, Western slopes high‐elevation of Mount Lebanon range, C4, Western slopes middle and low‐elevation of Mount Lebanon range, C5: North‐eastern Lebanon and Anti‐Lebanon mountains.

**TABLE 3:** Turnover and nestedness rates between paired global chorotypes in Lebanon.

	M	EM	IT	END	SA‐IT‐M	SA‐M	EM‐ES	EM‐IT	EM‐ES‐IT
M		0.58	1.00	0.90	1.00	0.99	0.97	0.66	0.88
EM	0.02		0.93	0.76	0.99	0.99	0.65	0.61	0.79
IT	0.00	0.09		0.70	0.99	1.00	1.00	0.91	0.95
END	0.06	0.16	0.07		1.00	1.00	0.86	0.66	0.72
SA‐IT‐M	0.04	0.19	0.04	0.00		1.00	1.00	0.97	0.99
SA‐M	0.31	0.22	0.00	0.00	0.00		1.00	1.00	1.00
EM‐ES	0.38	0.59	0.00	0.02	0.00	0.00		0.69	0.55
EM‐IT	0.02	0.17	0.01	0.24	0.09	0.00	0.13		0.85
EM‐ES‐IT	0.06	0.12	0.01	0.13	0.10	0.00	0.45	0.04	

*Note:* Values above the diagonal are Simpson turnover index and values below the diagonal correspond to nestedness (Sorensen dissimilarity minus Simpson turnover index).

Abbreviations: EM, East Mediterranean; EM‐ES‐IT, East Mediterranean, Euro‐Siberian, and Irano‐Turanian; END, Endemic to Lebanon; IT, Irano‐Turanian; IT‐EM, Irano‐Turanian and East Mediterranean; M, Mediterranean; M‐ES, Mediterranean and Euro‐Siberian; SA‐IT‐M, Saharo Arabian, Irano‐Turanian and Mediterranean; SA‐M, Saharo‐Arabian and Mediterranean.

When considering the local chorotype analysis, a similar scheme of species distribution partition is apparent in the country. The two species' richest chorotypes (C3 and C4) include more than half of all the species in this analysis (Table 2) and typically occur on the western side of Lebanon's mountain slopes (Figure 4). Likewise, there was an altitudinal stratification between these two chorotypes; the 24 species in chorotype C3 were restricted to the Mount Lebanon high elevation, while the 24 species in chorotype C4 were more frequent at lower altitudes and occupied the widest area of the country (Table 2). Two chorotypes occurred on the eastern side of the country. The chorotype C2, with only two species, occupied almost 20% of the country on the southeastern slopes of Lebanon and south Anti‐Lebanon, while the five species in C5 occupied about 15% of the country on the northeastern slopes of Lebanon and the Anti‐Lebanon range (Table 2, Figure 4). The chorotype C1 has the smallest surface, represented by a range‐restricted endemic to the southern Lebanon range. The turnover indices amongst regional chorotypes showed that the northwestern Lebanon and Anti‐Lebanon chorotype C5 reached maximum turnover when compared with the richer western Lebanon slopes, C3 and C4. Species with restricted or clumped distribution (e.g., *Quercus ithaburensis, Bupleurum fruticosum, Seartia tripartita*, and *Pistacia atlantica* ) were not grouped under any of the regional chorotypes.

### 3.4 Species Richness and Biogeographical Transition Zone

**FIGURE 5:**
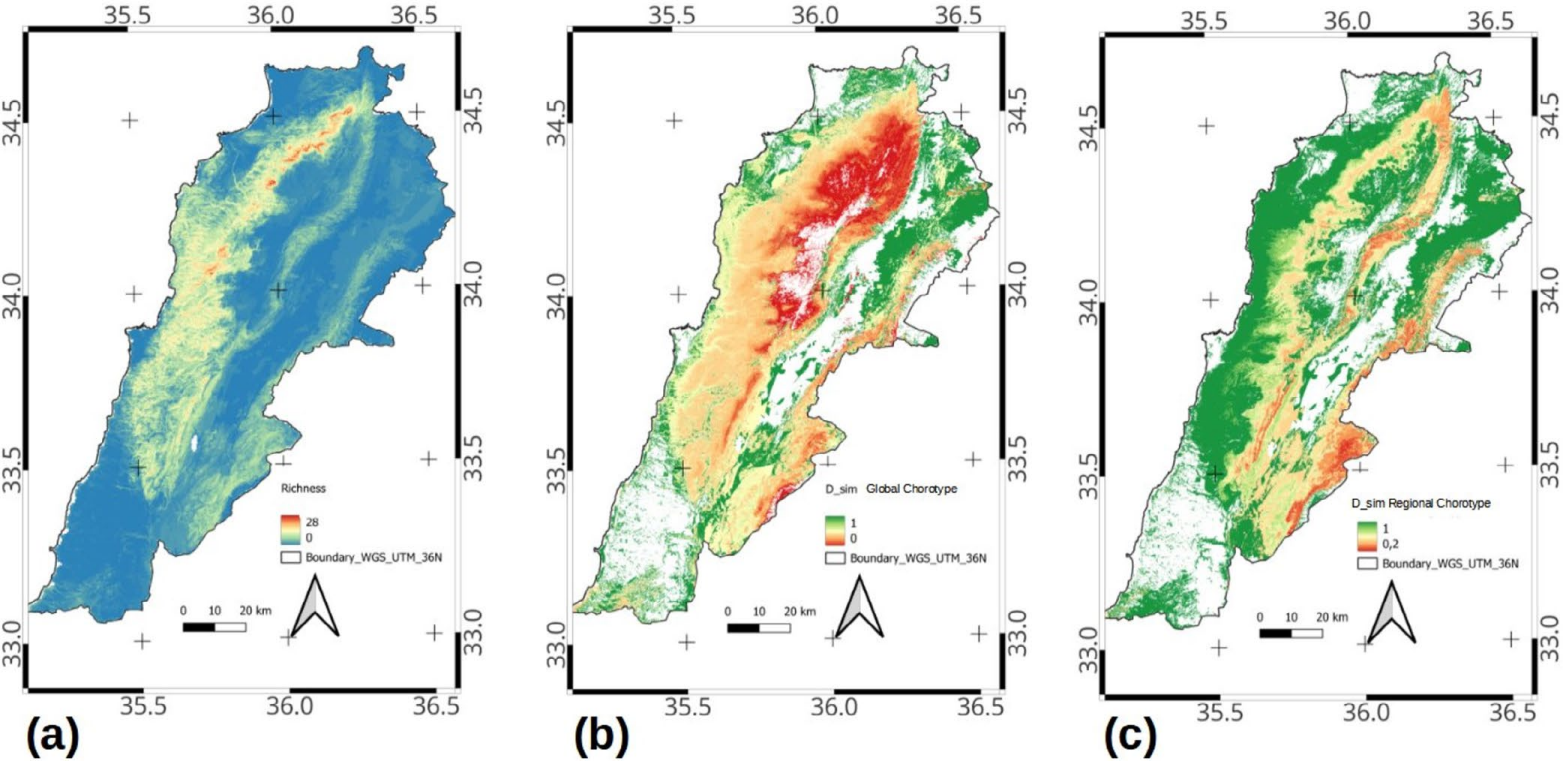
(a) Species richness gradients (b) The transition zone depicted by the mixture of species in each global chorotype (c) depicted by the mixture of species in each regional chorotype.

The species richness gradient resembled the distribution of the richer chorotypes. In particular, the Eastern Mediterranean global chorotype and the two regional chorotypes on the western slopes of Lebanon range C4 and C3. However, the hotspot of species richness occurred as a very narrow strip at intermediate altitude in the northwestern part of the mountain range (Figure 5a). This pattern seems to be due to the integration of the species‐rich global chorotypes Mediterranean and Eastern‐Mediterranean with species whose distribution range extends north to Euro‐Siberian regions, which occur at high elevations on the western slope of Mount Lebanon. When considering the regional chorotypes, only two chorotypes contribute to the species richness pattern: the western Lebanon mountain low to mid‐elevations (C4) and the mid‐elevation western slopes of Lebanon (C3). The biogeographical transition zone, measured as the degree of mixture amongst species belonging to different chorotypes, showed a different picture when compared to the species richness gradient. The most heterogeneous expanses, considering the global chorotypes patterns in tree and scrub communities, occurred at high elevation, above the richness hotspot, and extended southward and to the eastern slopes of Mount Lebanon, as well as to the southern parts of the Anti‐Lebanon range, particularly on Mount Hermon (Figure 5b). Then, the gradient of chorological heterogeneity continues towards the western middle elevation slopes of both the Lebanon and Anti‐Lebanon mountains and appears as an isolated spot in the southernmost lowlands of the country (Figure 5b). A rather similar pattern of chorological heterogeneity associated with the mountain ranges appeared when considering the mixture of species belonging to different regional chorotypes (Figure 5c). However, in this case, the maximum heterogeneity of chorotypes turned to the eastern slopes' higher elevation of Mount Lebanon and the western slopes of the Anti‐Lebanon range (Figure 5c). The gradient of chorological heterogeneity continues as a narrow strip around the mid‐elevation of Lebanon mountain range's western and southeastern slopes, as well as to the foothills of the southern Anti‐Lebanon range. The northern foothill of the Anti‐Lebanon range, the northern Beqaa valley, and the western foothill of the Lebanon range, as well as the coastal lowland, were rather homogenous considering the chorotypes pattern in tree and shrub communities (Figure 5c).

## 4 Discussion

**FIGURE 6:**
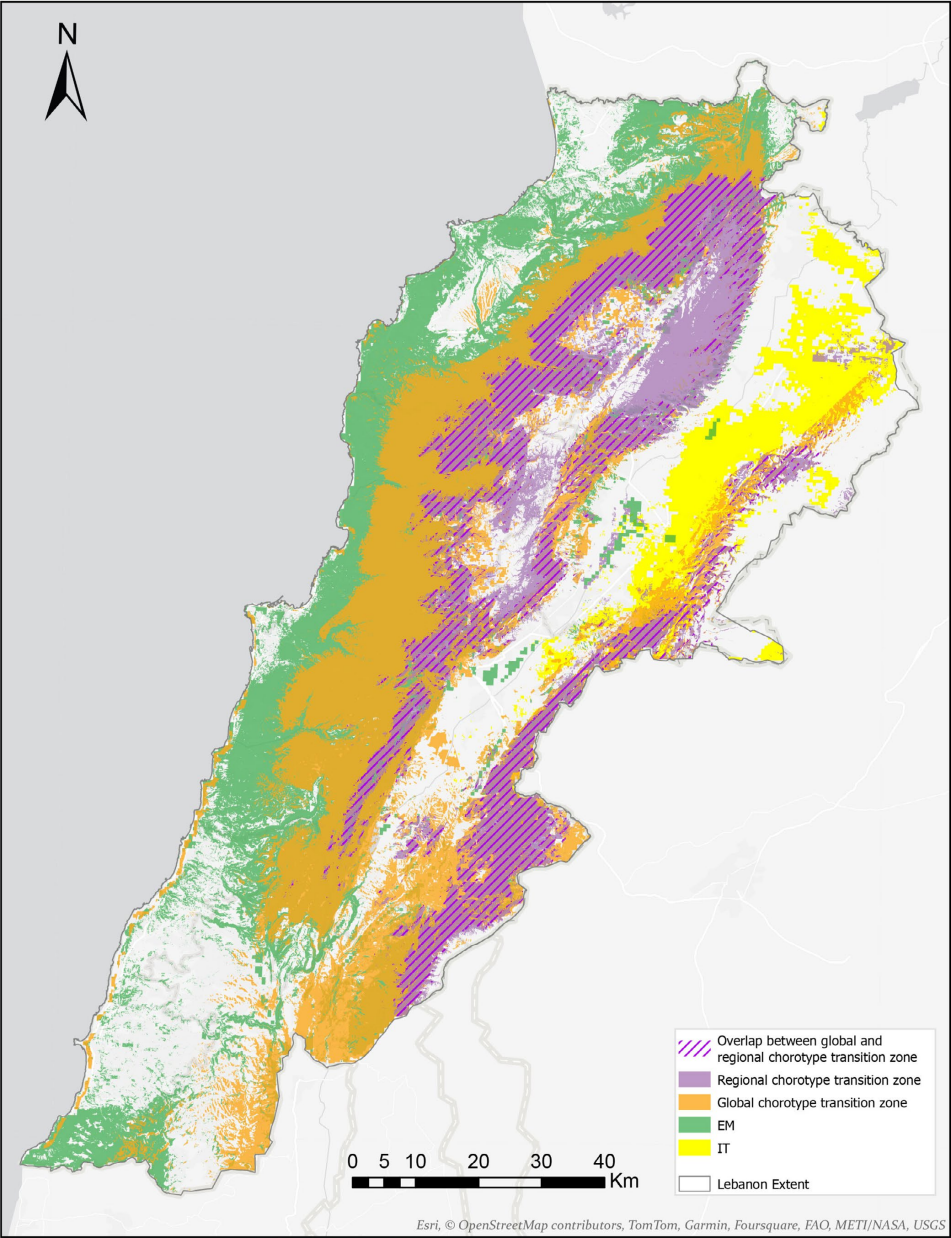
Lebanon biogeographical map.

In this study, we analyzed the biogeographic structure of Lebanon based on the distribution pattern of tree species. We used specimen and literature‐based locality records of the occurrence of trees and shrubs as input data for ecological niche modeling. Ecological niche modeling allows us to overcome the limitations of a grid system when there are numerous empty cells in the studied area because of scarce sampling records or transformed landscapes. For instance, within the Mediterranean region, land use is a mosaic of different patches, resulting in a high fragmentation of forests and natural ecosystems (Jomaa et al. 2009; Lenormand et al. 2018; Jomaa and Khater 2019). Thus, this method disables the barriers raised by solely relying on either climatic data or the occurrence of presence points by modeling both to produce a predicted spatial distribution of trees and shrub species as a surface on a map. The results of our modeled tree and shrub species cover most of the prevailing climax vegetation or head of a vegetation series that is the most spatially distributed and representative of the eastern Mediterranean basin and Asia (Barbero and Quézel 1989; Barbero et al. 1992; Browicz 1996). However, the generated maps integrate “blank” regions, which correspond to areas less suitable for species distribution (LOC below 50). These voids are areas exempt from trees, like high mountain steppe regions, or areas with unsuitable soil conditions (Figure 6). For instance, multiple climatic stressors determine the elevation range of tree species and the treeline, above which trees are replaced by scrubs (Bar‐On et al. 2022). Yet, possible reduction of such empty areas within the map could be achieved by including non‐phanerophytes dominant species in those formations, especially in steppe and high mountain areas, where phryganas composed of cushion‐like shrubs are prevailing (Ghazanfar and McDaniel 2016). Nonetheless, this type of vegetation characterized by a high level of endemism is worth an in‐depth analysis to determine its biogeographical affiliation.

The biogeographical affiliation of a taxon may vary depending on the observed attributes of the multiple aspects of life distribution on Earth and should be explicitly stated (Ferro 2024). In this analysis, we used floristically defined spatial units based solely on affinities in the geographical distribution of taxa, with no evolutionary nor temporal dimensions considered (See Morrone 2014; Passalacqua 2015; Fattorini 2016, for discussion on concepts and terms). Thus, we rely on species geographical alliances, or chorotypes, to characterize Lebanon phanerophytes biogeography. However, as suggested by Fattorini (2015) we differentiated between global chorotypes, the whole range of species distribution exceeding our studied area, and regional chorotypes, local within Lebanon with similar species distribution. The former may represent worldwide spatial responses of species to historical and environmental pressures, while the latter represents species assemblages with local ecological conditions. Our results revealed a similar biogeographic structure within Lebanon, considering both local and global chorotypes, with most species clumped on the western side of Mount Lebanon and a few species eastwards of the Beqaa valley. The two western regional chorotypes may be divided into lowland (C4) and highland (C3) assemblages, including species belonging to the five global chorotypes (Table 1). However, there is a gradual loss of pure Mediterranean species with elevation and distance from the sea (Stephan et al. 2020), with replacement by vicariant species deriving from speciation under changing environmental factors (Anacker and Strauss 2014). The highland regional chorotype C3 mainly includes those species whose distribution extends northwards from the Mediterranean into the Euro‐Siberian region and Irano‐Turanian at higher altitudes along the tree line, especially on the north‐western slopes of Mount Lebanon (Table 1, Figure 3). The cool and mesic conditions of high altitude on the western slopes favor the subsistence of species extending into the Euro‐Siberian region as relicts in biodiversity refugium of the glaciation period (Médail and Diadema 2009; Fady and Conord 2010). However, deforestation and land degradation enhanced the intrusion of Irano‐Turanian elements within the East Mediterranean, especially in the middle elevations and southern Mount Lebanon and further south into Palestine (Zohary 1973; Abi Saleh et al. 1996).

The restriction of most species in our analysis to the western side of the Lebanon Mountain reduces species richness eastwards, in the Beqaa valley and Anti‐Lebanon range, probably due to harsh climatic conditions coupled with historical and persisting human activities that have shaped tree diversity (Mouterde 1966; Asouti and Kabukcu 2014; Asouti et al. 2015; Noroozi and Körner 2018). Despite the impoverished tree richness, eastern Lebanon is distinct considering floristic spatial units. Indeed, the turnover (β_sim) and nestedness (β_nest) indices for pairwise surface comparison amongst regional chorotypes reached the highest turnover and lowest nestedness values for the northeastern chorotype C5 when compared with the species‐rich western Lebanon C3 and C4 regional chorotypes (Table 4). Similarly, the northeastern Irano‐Turanian global chorotype reached high values of turnover and low nestedness when compared with the other global chorotypes (Table 3). Although there are no causal assumptions in our analysis, the limited nestedness and high turnover rates for both the western chorotypes (e.g., East‐ and Mediterranean) and the eastern Irano‐Turanian may be explained by an aridity gradient amplified by highly human‐induced land legacy dynamics since the Holocene (Hajar et al. 2008; Saatkamp et al. 2020; Cruz‐Alonso et al. 2021). A recent extensive analysis of tree distribution has found a strong association between tree range edges and pronounced spatial climatic gradients, suggesting biome edges to be strong delineators of tree distributions (Lerner et al. 2023).

**TABLE 4:** Turnover and nestedness rates between paired regional chorotypes in Lebanon.

	C1	C2	C3	C4	C5
C1		0.52	0.20	0.45	0.18
C2	0.40		0.47	0.49	0.16
C3	0.61	0.47		0.59	0.81
C4	0.24	0.48	0.11		0.75
C5	0.65	0.82	0.03	0.11	

*Note:* Values above the diagonal are Simpson turnover index and values below the diagonal correspond to nestedness (Sorensen dissimilarity minus Simpson turnover index). C1: Southern Mount Lebanon range C2: Southeastern Lebanon and Anti‐Lebanon mountains, C3: Western slopes high‐elevation of Mount Lebanon range, C4: Western slopes middle and low‐elevation of Mount Lebanon range, C5: North‐eastern Lebanon and Anti‐Lebanon mountains.

Beyond the hypothetical causes of the observed pattern, our results on floristic spatial unit turnover and nestedness suggest a clear biogeographic structure. The west of Mount Lebanon can be assigned to the (East) Mediterranean region, with higher altitude enclaves dominated by species distributed northward and eastward into the Euro‐Siberian and Irano‐Turanian regions respectively, while the highly dissimilar northern Beqaa and Anti‐Lebanon belong to the Irano‐Turanian region (Figure 6).

It is worth mentioning that both Saharo‐Arabian and East Mediterranean global chorotypes also reached the highest turnover and lowest nestedness values (Table 3). However, these global chorotypes occupy very small marginal areas in southern Lebanon and only include one species each (Table 2). *Searsia tripartita*, attributed to the Saharo‐Arabian, is strictly observed on the southern coastline (Figure 3i) and may suggest the presence of facies attributed to the inframediterranean vegetation level *sensu* Blondel et al. (2010). Similarly, *Pistacia atlantica* , an Irano‐Turanian element extending into the Mediterranean and the Saharo‐Arabian (Figure 3h), thrives in the Rift Valley facies from Aqaba to the Jourdan River basin (Zohary 1973; Danin 1988; Blondel et al. 2010). Note that neither *Pistacia atlantica* nor *Searsia tripartita* were included in any regional chorotype, suggesting the necessity for a more detailed analysis of plant communities in those areas. Similarly, *Quercus ithaburensis* and *Bupleurum fruticosum* (assigned to East and Mediterranean global chorotypes respectively) were not assigned to any regional chorotype, probably due to their restricted distribution in Lebanon. *Quercus ithaburensis* occurs at low altitudes in the extreme north or extreme south in the rift valley, while *Bupleurum fruticosum* has a very clumped distribution restricted to small areas. Finally, a drawback of our analysis for this southern region is the exclusion of species with too few records to generate accurate, valid predicted areas of distribution through ecological niche modeling. Species with Saharo‐Arabian distributions, such as *Retama raetam* on the coast and *Ziziphus spina‐christi* present in the Rift Valley, were not included in our analysis and thus precluded a more robust generalization of Saharo‐Arabian regional chorotype distribution in Lebanon.

The recognition of range‐restricted endemic species occurring in rather small geographical areas is an essential input for conservation biology. We recognized three endemic phanerophyte species: *Quercus kotschyana, Quercus look*, and *Pyrus bovei*, and grouped them into the Endemic global chorotype. The three endemic species follow a distribution pattern related to the Lebanon mountainsides (Figure 3e). However, their distribution within Lebanon does not match perfectly, as noted by the regional chorotype analysis (Table 1). Only one species, *Quercus look*, endemic to southern Lebanon and the Anti‐Lebanon mountain ranges, was distinguished in a regional chorotype C1 (Figure 4). *Quercus kotschyana*, an endemic species inhabiting the northwestern slopes of Mount Lebanon, was included in the western slopes of the high elevation of Mount Lebanon chorotype C3 (Figure 4). Finally, *Pyrus bovei* is an endemic species occurring in the northeastern slopes of the Mount Lebanon range and the northern Anti‐Lebanon range, thus included in the northeastern Lebanon and Anti‐Lebanon mountains C5. Even when the three Lebanon endemics are not spatially congruent, their distribution matches the location of the sharpest biogeographical transition zone, as shown by the mixture pattern of both the global and regional chorotypes (Figure 4b,c). Such distribution of endemic species within transitional zones at high altitudes converges with the findings of other researchers within the Mediterranean basin (Fenu et al. 2014; Abdelaal et al. 2020).

Acknowledging that biogeographical transition zones involve areas of juxtaposition, in red, between more homogeneous biogeographic units, in green (Figure 5b,c); our results indicate the eastern Lebanon lowland as part of the (East)Mediterranean region while the northern Beqaa and northern tip of the Anti‐Lebanon range lie in the Irano‐Turanian region (Figure 6). Thus, the country is dominated by species belonging to the Mediterranean and Eastern Mediterranean considering species richness and occupied surface. Mount Lebanon constitutes an apparent barrier for species in the Mediterranean chorotype that do not extend eastwards, as well as for those in the eastern Irano‐Turanian region. The breadth of the transition zones, as defined by a gradient of species mixture from different regions, may vary depending on the nature of the studied system and the degree of mixture considered. In Figure 5b, the transition is large, considering the two higher intervals of the mixture gradient (drawn in red and orange). This is not surprising since mountain ranges frequently exhibit juxtapositions of species with different geographical affinities. However, if we consider only the highest interval of species mixture (colored only in red), the transition zone is much more restricted, particularly to the higher elevation of the mountains where the tree diversity drops in the alpine zone above the tree line. Similarly, the eastern slopes in the rain shadow and most of the Anti‐Lebanon range are therefore tree impoverished (Figure 5a), indicating a subtraction biogeographical transitional zone (Figure 6). The few Lebanon endemic trees are also considered characteristics of these transition zones.

## Author Contributions

**Jean Stephan:** conceptualization (lead), data curation (equal), formal analysis (lead), investigation (equal), methodology (equal), project administration (lead), supervision (lead), validation (lead), writing – original draft (lead). **Youmna Hammoud:** resources (equal), software (equal), visualization (equal). **Melissa Korban:** formal analysis (equal), resources (equal), software (equal), visualization (equal). **Ignacio Ferro:** data curation (equal), formal analysis (equal), investigation (equal), methodology (equal), software (equal), visualization (equal), writing – review and editing (equal).

## Conflicts of Interest

The authors declare no conflicts of interest.

## Supporting information


Data S1.

## Data Availability

Location records and environmental variables used to generate the models are made available on Dryad (https://doi.org/10.5061/dryad.hmgqnk9vf). Some of the content has already been published by Stephan et al. in 2020, Hammoud and Stephan in 2022, and Stephan and Korban in 2025.

## Appendix A

**TABLE A1:** Predictors: Data source and calculation tools.

Code	Predictor	Source	Tool
DEM	Digital elevation model (m)	Contour line^a^	Topo to raster
Aspect	Aspect (°)	DEM	Aspect
Slope	Slope (%)	DEM	Slope
DFS	Distance from the sea (m)	DEM	Fishnet—Near
CC	Mean cloud coverage (May–July) (%)	Cloud Raster^b^	Mean calculation
Tmin	Mean of minimal temperature of the coldest month (°C)	Mean of minimal temperature of the coldest month^c^	Mean calculation
Tmax	Mean of maximum temperature of the hottest month (°C)	Mean of maximum temperature of the hottest month^c^	Mean calculation
P	Cumulative annual precipitation (mm)	Precipitation Isohyets^d^	—
EQ	Emberger quotient	Cumulative annual precipitation	Raster calculator
		Mean minimal temperature of the coldest month	
		Mean maximal temperature of the hottest month	
AWC	Available water content (mm)	Soil maps	—
Flow	Flow accumulation	DEM	Fill; flow direction; flow accumulation
NDVI	Normalized Difference Vegetation Index	Mosaic of Landsat 8‐operational land imager images	Raster calculator
LST	Land surface temperature (°C) average for summer months	Mosaic of Landsat 8 Images	Raster calculator
LD	Land degradation	Land productivity^e^	SDG Land degradation plugin
		Land cover^e^	
		Soil organic carbon^e^	
Soil type	Geze soil	Geze classification^f^	Feature to Raster

^a^ The Lebanese Army Geographic Department.

^b^ National Scientific Research Center (2002–2013).

^c^ WorldClim (1970–2000).

^d^ National Scientific Research Center.

^e^ Trends.Earth Data, Sustainable Development Goals Plugin.

^f^ National Scientific Research Center, Soil Maps.
